# RNAi Methodologies for the Functional Study of Signaling Molecules

**DOI:** 10.1371/journal.pone.0004559

**Published:** 2009-02-24

**Authors:** Gwang Lee, Leah A. Santat, Mi Sook Chang, Sangdun Choi

**Affiliations:** 1 Department of Molecular Science and Technology, College of Natural Science, Ajou University, Suwon, Korea; 2 Institute for Medical Sciences, College of Natural Science, Ajou University, Suwon, Korea; 3 Molecular Biology Laboratory, Alliance for Cellular Signaling, Division of Biology, California Institute of Technology, Pasadena, California, United States of America; 4 Department of Biological Sciences, College of Natural Science, Ajou University, Suwon, Korea; Massachusetts General Hospital/Harvard University, United States of America

## Abstract

RNA interference (RNAi) was investigated with the aim of achieving gene silencing with diverse RNAi platforms that include small interfering RNA (siRNA), short hairpin RNA (shRNA) and antisense oligonucleotides (ASO). Different versions of each system were used to silence the expression of specific subunits of the heterotrimeric signal transducing G-proteins, G alpha i2 and G beta 2, in the RAW 264.7 murine macrophage cell line. The specificity of the different RNA interference (RNAi) platforms was assessed by DNA microarray analysis. Reliable RNAi methodologies against the genes of interest were then developed and applied to functional studies of signaling networks. This study demonstrates a successful knockdown of target genes and shows the potential of RNAi for use in functional studies of signaling molecules.

## Introduction

RNA interference (RNAi) has been widely used by animal cell biologists as a gene silencing tool to study the cellular effects generated by modulating the expression of individual genes [1], [2], [3], [4], [5], [6]. Expression can be reduced by introducing gene-specific double-stranded RNA (dsRNA) or single-stranded RNA (ssRNA) into a cell. Subsequently, the small RNA products generated from Drosha and/or Dicer-mediated dsRNA processing are delivered to the RNA-induced silencing complex (RISC), which is implicated in mRNA destruction and translational repression, and the RNA-induced transcriptional silencing complex (RITS), which is implicated in chromatin silencing [7], [8], [9].

However, difficulties remain in finding the best way to make RNAi work as a gene-specific silencing procedure for signaling studies. For example, dsRNA [10] or ssRNA [11], [12], [13] can stimulate innate cytokine responses in mammals. Additionally, synthetic small interfering RNAs (siRNAs) formulated in nonviral delivery vehicles can be potent inducers of interferons and inflammatory cytokines in mice and humans [14]. siRNAs can also mediate sequence-independent gene suppression and induce immune activation by signaling through toll-like receptor 3 (TLR3) [15]. A recent study showed that dsRNAs that are 21 nucleotides or longer are also involved in anti-angiogenic responses [16]. These could significantly limit the application of RNAi and result in off-target effects and immuno-stimulation associated with the nucleic acid treatments. In order to develop reliable RNAi reagents, three different platforms were tested: small interfering RNA (siRNA), antisense oligonucleotide (ASO) and short hairpin RNA (shRNA). siRNA, ASO and shRNA were designed to knockdown the endogenous G protein alpha i2 (Gαi2) or G protein beta 2 (Gβ2) gene in screens in RAW 264.7 murine macrophage-like cells. A series of gene expression profiling experiments and quantitative reverse transcriptase-polymerase chain reaction (QRT-PCR) analyses were carried out to assess both the specificity of the target gene knockdown and the functional studies of the targeted signaling molecules. Gαi2 knockdown cells were tested to monitor the changes in signaling networks after treatment with different ligands, in this case lipopolysaccharide (LPS), Pam_2_CSK4 (Pam_2_) or prostaglandin E2 (PGE_2_).

## Results

### DNA Microarray and RT-PCR verification of expression of specific genes in RAW 264.7 cells

To develop a clear picture of the nature of the genes expressed in RAW 264.7 cells, Affymetrix GeneChip screening was performed, producing duplicate array data sets. Affymetrix Mouse 430 chips A and B were used to assess the gene expression of RAW 264.7 cells, mouse bone marrow derived macrophage (BMDM) cells and the mouse macrophage cell line, J774A.1. BMDM and the cell lines have ‘Present Call’ gene elements ranging from 17K to 19K (data available at The Signaling Gateway: http://www.signaling-gateway.org/data/micro/cgi-bin/micro.cgi?exptaffy). Table 1 shows that a gene marked ‘Present’ in the Affymetrix GeneChip data was almost always determined to be ‘Present’ when measured using RT-PCR. To avoid any possible errors in the design of PCR primer sets, genes showing an ‘Absent’ signal in RT-PCR during the first trial were verified using 2 to 3 alternative primer sets. Overall, approximately 5% of the transcripts found to be present in the microarray were absent according to RT-PCR, and 30–40% of absent determinations according to the microarray were detected by RT-PCR (Table S1). However, in cases in which multiple probes existed on the chip (38% of the probes), reproducible calls (P: Present or A: Absent) improve the odds of a correct result. The presence of Gαi2 and Gβ2 mRNAs was verified using a DNA microarray and by a RT-PCR analysis (Table 1).

**Table 1:** Expression of heterotrimeric G protein subunits and RGS proteins in RAW 264.7 cells, as assessed by Affymetrix GeneChips and RT-PCR (P: present, A: absent).

Symbol	Gene Name	Affymetrix Calls	RT-PCR Results	Abundance	UMRR*
Gnai1	G alpha i1 subunit	A	A		P
Gnai2	G alpha i2 subunit	P	P	high	
Gnai3	G alpha i3 subunit	P	P	medium	
Gna11	G alpha 11 subunit	P	P	medium	
Gna14	G alpha 14 subunit	A,A	A		P
Gna15	G alpha 15/16 subunit	A	P	high	
Gnaq	G alpha q subunit	P	P	high	
Gnb1	G beta 1 subunit	P,A,P	P	high	
Gnb2	G beta 2 subunit	P	P	medium	
Gnb3	G beta 3 subunit	A	A		P
Gnb4	G beta 4 subunit	P	P	medium	
Gnb5	G beta 5 subunit	A,P	P	high	
Gng2	G gamma 2 subunit	P,A,P	P	low	
Gng3	G gamma 3 subunit	A	A		P
Gng4	G gamma 4 subunit	A,A,A	A		A
Gng5	G gamma 5 subunit	P,P,A	P	medium	
Gng7	G gamma 7 subunit	A	P	low	P
Gng8	G gamma 8 subunit	A	P	medium	
Gng10	G gamma 10 subunit	P			
Gng11	G gamma 11 subunit	A	P	medium	
Gng12	G gamma 12 subunit	P			
Gng13	G gamma 13 subunit	A	A		P
Gng14	G gamma 14 subunit	P			
Rgs4	regulator of G protein signaling 4	A,A,A	A		P
Rgs7	regulator of G protein signaling 7	A	A		P
Rgs8	regulator of G protein signaling 8		P	medium	P
Rgs13	regulator of G protein signaling 13	A	P	low	P
Rgs16	regulator of G protein signaling 16	A,A,A	P	high	
Rgs19	regulator of G protein signaling 19	P,P	P	high	P

* RT-PCR results in Universal Mouse Reference RNA (11 cell line mixture).

### Establishment of cells lacking Gαi2 and Gβ2 proteins

G protein coupled receptors are characterized by seven transmembrane domains, and ligands stimulating these receptors are diverse and include immuno-stimulating molecules. Heterotrimeric G proteins are composed of α, β and γ subunits, with the G protein alpha subunits consisting of four families: Gαi/o, Gαs, Gαq/11 and Gα12/13. The Gαi family is further characterized into Gαi1, Gαi2 and Gαi3. Gαi2 has been reported to inhibit the activities of types I, V, and VI adenylyl cyclase isoforms directly [17]. As Gαi2 appears to be involved in a complex pattern of signaling [17], [18], [19], [20], [21], [22], [23], [24], [25], [26], [27], insight into its function was sought by silencing the Gαi2 transcript. In addition, the Gβ2 subunits released upon activation of the heterotrimeric G protein activate specific effectors, and previous studies have shown it to be the primary beta subunit for certain Gαi signaling pathways in macrophage cells [5], [6]. Thus, Gβ2 was also targeted to test the RNAi mechanisms.

**Figure 1:**
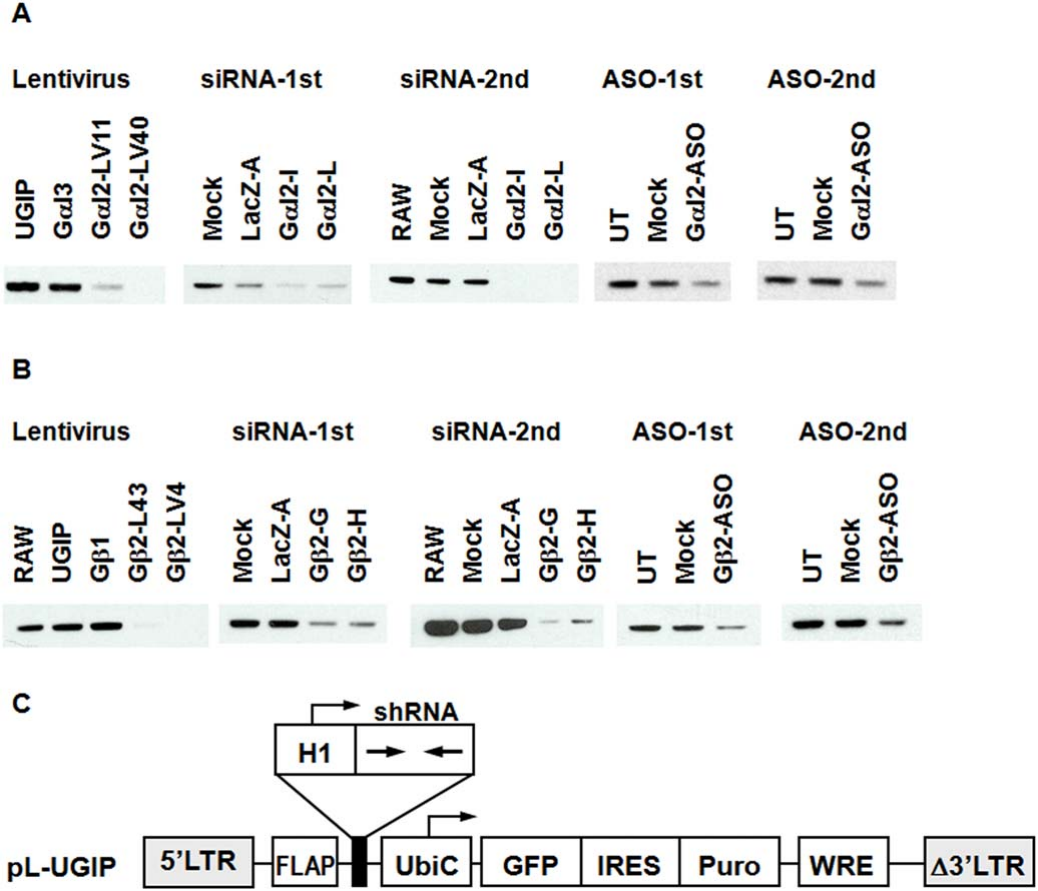
Western blot analysis of (A) Gαi2 and (B) Gβ2 proteins in shRNA, siRNA and ASO treated RAW 264.7 cells. Two independent experiments are shown for the siRNA and ASO treatments. Blots from the shRNA-expressing lentiviral (LV) lines are representative of multiple samples taken over a 5-week period. Significant target gene knockdown is observed with all three platforms. UGIP: control lentivirus transfected cell line, UT: untreated, Mock: mock-treated. (C) Schematic diagram of the lentiviral construct used to generate shRNA expressing RAW 264.7 cell lines. The hairpin form of siRNA is expressed under the control of a mouse H1RNA polymerase III promoter. The vector also contains the enhanced GFP marker gene and the puromycin resistance gene (Puro) regulated by a UbiC promoter. IRES, internal ribosome entry site; FLAP, HIV-1 FLAP element; WRE, woodchuck hepatitis post- transcriptional regulatory element.

RAW 264.7 cells were transfected with siRNA or ASO against Gαi2 or Gβ2. Western blot results (Figures 1A and B) showed a significant knockdown of the target gene in each of experiments. RAW cells were transfected using FuGENE6 (Roche Molecular Biochemicals, Indianapolis, IN) for ASO and Lipofectamine 2000 (Invitrogen, Carlsbad, CA) for siRNA. Cells were harvested at 72 hr post-transfection.

Pseudotyped lentivirus carrying shRNA can be efficiently integrated into the chromosome, making it a good carrier for the delivery and sustained expression of shRNA [5], [28]. Lentiviral vectors were constructed with expression cassettes that allowed the expression of antibiotic selection markers (e.g., puromycin) in order to identify and enrich the fraction of transduced cells (Figure 1C). Cells were infected with a lentivirus containing a previously validated Gαi2 or Gβ2 shRNA [5], [6]. The target gene silencing activity of each shRNA in RAW264.7 cells was assessed by QRT-PCR and immunoblotting for endogenous proteins. Consistent with the QRT-PCR data, significant decreases in the levels of Gαi2 or Gβ2 proteins were observed in the shRNA expressing cells (Figure 1).

### siRNA, ASO and shRNA as RNAi tools

**Figure 2:**
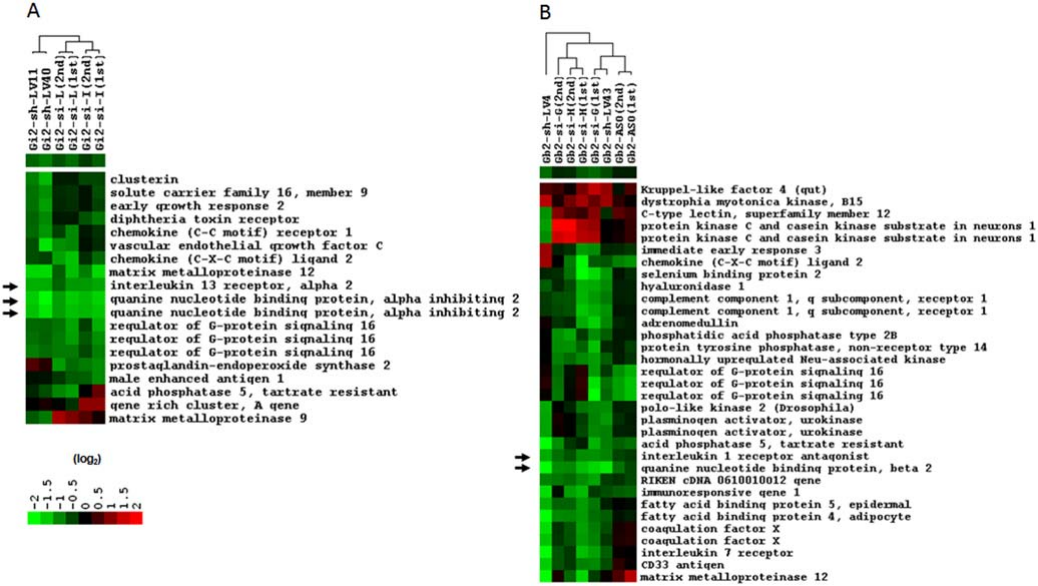
Hierarchically clustered dendrograms of gene expression changes. Clustering was achieved using the CLUSTER and TREEVIEW programs (http://rana.lbl.gov/EisenSoftware.htm). Each row represents a gene, and each column represents a particular sample. (A) Gαi2 gene knockdown (LV40 and LV11: shRNA- expressing lentivirus transfected cell lines; I and L: I or L form of siRNA transfected into cells). (B) Gβ2 gene knockdown (LV4 and LV43: shRNA-expressing lentivirus transfected cell lines; G and H: G or H form of siRNA transfected into cells; Gb2-ASO: antisense oligonucleotide). The expression level relative to that of the control cells is provided by colors shown in log_2_ scale. The target genes, Gαi2 and Gβ2, are highlighted by arrows. The apparent down-regulation of IL13ra2 in the Gαi2-deficient cells and IL1rn in the Gβ2-deficient cells are also highlighted (see context).

It was confirmed by DNA microarray analysis using custom-made inkjet-printed 16K oligonucleotide chips (Gene Expression Omnibus platform accession number GPL254: http://www.ncbi.nlm.nih.gov/projects/geo/) that the transfection of siRNA against Gαi2 or Gβ2 showed significant levels of the target gene knockdown (Figure 2). Cells were harvested at 48 hr post-transfection for assessment of mRNA, and gene expression was assessed using a DNA microarray and by QRT-PCR. Control samples were mock-treated with transfection reagent to determine the baseline levels of gene expression. It was found that 400 nM siRNA is invariably more effective than 100 nM or 200 nM. Although several papers have noted that siRNA reaches maximal effectiveness at a concentration lower than 100 nM [29], [30], Song et al. [31] reported that primary macrophages required 1 µM siRNA to achieve a maximal effect. For any given siRNA, a comparable level of knockdown can be achieved with at least a 10-fold lower siRNA concentration using NIH3T3 cells (data not shown). The present results suggest that the requirement for a high siRNA concentration in RAW264.7 cells is simply a function of their low transfection efficiency.

Validated ASOs against the Gαi2 or Gβ2 gene were obtained from ISIS Pharmaceuticals, Inc. (Carlsbad, CA). The ASOs used were phosphorothioate oligodeoxynucleotides with 2′-O-methoxyethyl incorporated to enhance their affinity for RNA sequences and their resistance to degradation by nucleases. RAW 264.7 cells were transfected with 400 nM of ASO and were harvested for mRNA analysis at 48 hr post-transfection. Results from the DNA microarrays showed a detectable knockdown for ASO against Gβ2 message (Figure 2B) as well as a specific knockdown for ASO targeting Gαi2. Another set of Gαi2 ASO data was not included in the analysis shown in Figure 2A due to the unexpected induction of TLR-related genes. This will be discussed later.

It has been shown that the knockdown effect mediated by siRNA or ASO is sustained for up to several days [29], [32], [33]. However, creation of a cell line infected with a shRNA-expressing lentivirus provides an approach for gene knockdown over a longer term. The analysis in Figure 2 shows that lentiviral vector-based RNAi works well in RAW 264.7 cells with a robust knockdown of the Gαi2 and Gβ2 target genes.

### Induction of TLR-related genes by ASO designed for a knockdown of Gαi2

**Figure 3:**
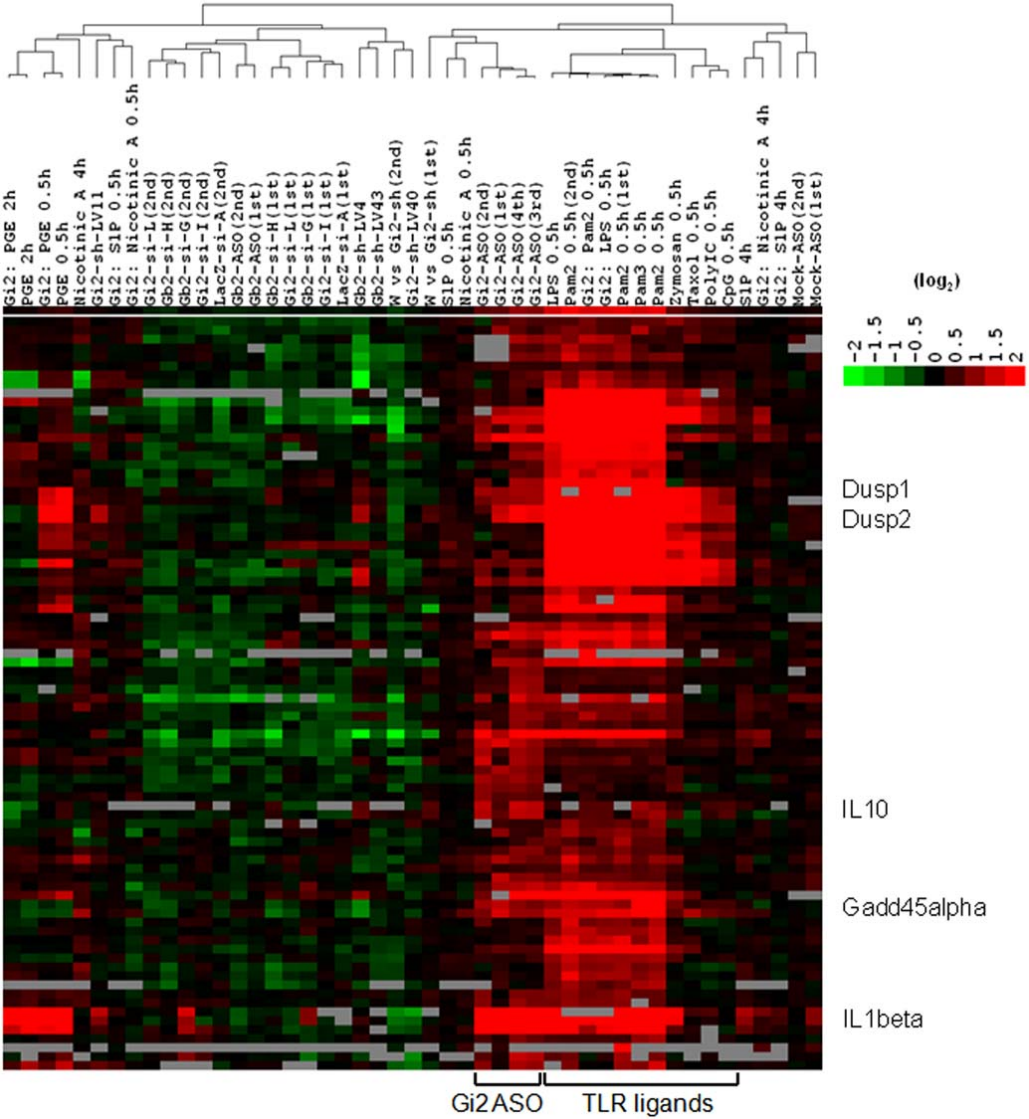
Hierarchical clustering of gene expression for different RNAi platforms or ligand treatments in Gαi2 knockdown RAW 264.7 cells. While Pam_2_ 0.5 h (1st) and Pam_2_ 0.5 h (2nd) were tested in lentivirus vector transfected RAW cell lines, Pam_2_ 0.5 h was carried out in wild type RAW 264.7 cells together with other TLR ligand experiments, in this case Pam_3_ , Zymosan, Taxol, PolyIC and CpG.

The ASO tests showed an unexpected observation of a significant degree of induction of TLR-related genes by one of ASOs designed for the knockdown of the Gαi2 gene. Kim et al. [34] found a very potent induction of interferon α and β by short single-stranded RNAs (ssRNAs) transcribed with T3, T7 and Sp6 RNA polymerases. In their studies, analyses of the potential mediators of this response revealed that the initiating 5′ triphosphate is required for interferon induction. Moreover, single-strand RNA bearing 5′ phosphate could activate RIG-1 mediated anti-viral responses [35] and it was also shown that 5′ triphosphate is the ligand for RIG-1 [36]. However, with the present ASO, neither RNA polymerase nor 5′ triphosphate was used. Nonetheless, a transcription profiling analysis revealed patterns of regulation that were very similar to the patterns of expression induced by ligands that trigger TLRs and clustered with them (Figure 3), showing a higher expression of IL1β, IFNα-inducible protein, chemokine (C-C motif) ligand 2 (Ccl2), IFN-induced protein with tetratricopeptide repeats 1 (Ifit1), 2 (Ifit2) and 3 (Ifit3), granulocyte colony stimulating factor 3 (Csf3), immunoresponsive gene 1 (Irg1), IL6, guanylate nucleotide binding protein 1 (Gbp1), 2 (Gbp2) and 4 (Gbp4), dual specificity phosphatase 1 (Dusp1) and 2 (Dusp2), IL10, and growth arrest and DNA-damage-inducible 45α (Gadd45α) (not all data shown: separate paper in preparation). When the ASO sequence for Gαi2 was studied further, it was found that the Gαi2 antisense oligonucleotide sequence has several CpG motifs (5′-TTT AGA G**CG** CT**C G**GC TGC **CG**-3′: multiple unmethylated **CpG** appeared and one purine-purine-**CpG**-pyrimidine-pyrimidine existed). Unmethylated CpG motifs are present in bacterial genomic DNA and function as a pattern recognition motif by the host innate immune system [37], [38]. For mouse, the sequence GA**CG**TT appears to be optimal, but flanking nucleotides to the central CpG core appear to influence recognition to some degree. Oligonucleotides containing the core CpG motif might bind to the TLR9 whose expression was confirmed in RAW 264.7 cells (Table 2). TLR9 engagement triggers alteration of the cellular redox balance, tyrosine phosphorylation of vav1 by a src-related tyrosine kinase [39], and the induction of cell signaling pathways including mitogen-activated protein kinases (MAPKs) and NFκB [37]. Interleukin-1 receptor-associated kinase (IRAK) and/or tumor-necrosis-factor-receptor-associated factor 6 (TRAF6) may be diverging points for NFκB activation in response to CpG DNA in RAW264.7 cells [40], [41]. In our ASO sequence against Gαi2, three copies of the unmethylated CpG motif per oligonucleotide led to the enhanced activation of TLRs.

**Table 2:** RT-PCR confirmation of TLR expression in RAW 264.7 cells (P: present, A: absent).

Gene Name	RT-PCR Results in RAW	Abundance	Affymetrix Chip Call
TLR1	P	High	P
TLR2	P	High	P
TLR3	P	High	P
TLR4	P	Low	P
TLR5	A		A
TLR6	P	Medium	P
TLR7	P	High	P
TLR8	A		A
TLR9	P	High	A
TLR10 (not in mouse)			
TLR11	A		
TLR12	P	Medium	
TLR13	P	High	

### The effects of Gαi2 or Gβ2 knockdown

Gene expression analyses were performed for all of the transduced cells using custom-made 16K oligonucleotide DNA microarrays. It is well known that the individual members of the G protein heterotrimers are necessary for the stability of their binding partners [5], [42]. While the regulated number of genes in each knockdown experiment in which a two-fold cut-off was used was between about 30 to 80, changes in the mRNA expression levels of other α, β or γ G proteins were not detected in siRNA- or ASO-treated cells or in shRNA-containing cells (Table S2), indicating that the absence of the Gαi2 or Gβ2 subunit protein did not affect the levels of transcripts encoding other individual members of the G protein heterotrimers.

**Figure 4:**
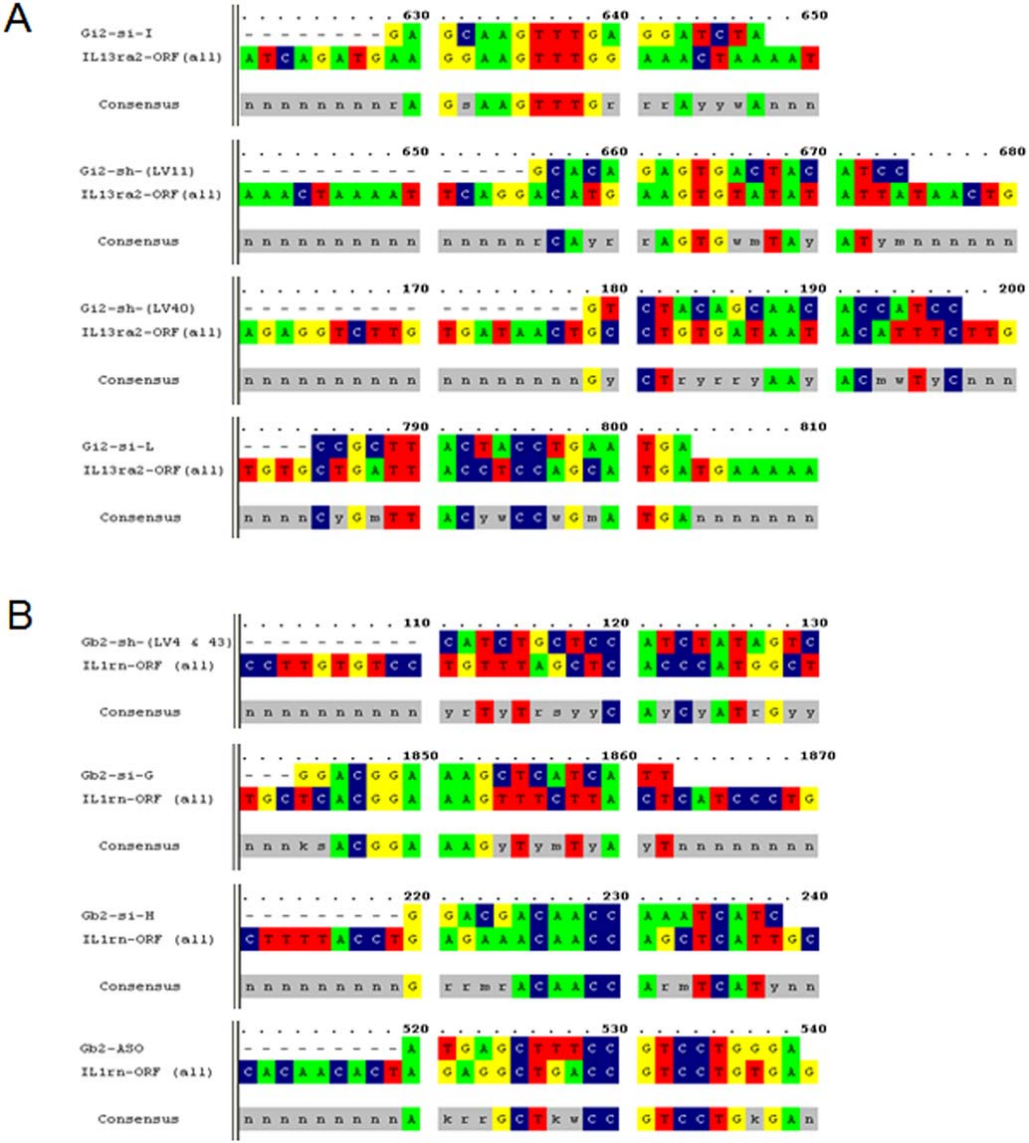
Sequence homologies of the oligonucleotides used in siRNA, shRNA or ASO for (A) Gαi2 or (B) Gβ2. Sequences were analyzed by OMIGA 2.0 (Rainbow Technologies, Inc.).

In addition to the knockdown of the intended target, apparent regulation of other genes depending on the RNAi platforms was found (Figure 2). As shown in Figure 2, there appeared to be up- or down-regulations of some genes in both Gαi2- and Gβ2-deficient cell lines. The apparent down-regulation of interleukin 13 receptor alpha2 (IL13ra2) in the Gαi2-deficient cells and interleukin 1 receptor antagonist (IL1rn) in the Gβ2-deficient cells may be noteworthy, as these changes were observed with all platforms (Figure 2). This result is intriguing given the fact that these proteins are not reported as being capable of coupling to either Gαi2 or Gβ2. A biological link was not found for Gαi2 or Gβ2 to IL13ra2 or IL1rn in published reports; however, it was noted that the selected oligonucleotide sequences used for Gαi2 or Gβ2 RNAi tests had homology of 7 to 8 contiguous nucleotides to either IL13ra2 (siRNA structure I) or IL1rn (siRNA structure G and ASO) (Figures 4A and B). While the direct silencing of nontargeted genes containing as few as eleven contiguous nucleotides of identity to the siRNA has been reported [43], it appears to be unlikely that 7 to 8 contiguous nucleotides of identity can regulate the expression of unintended targets. Furthermore, given the fact that not all of the siRNA, shRNA or ASO sequences showed significant homology to the IL13ra2 or IL1rn sequences (Figure 4), these common down-regulations may be due to an unappreciated biological consequence of Gαi2 or Gβ2 depletion and shed light onto the unidentified functions of these G-protein subunits. Interestingly, the expression of RGS16 (Regulators of G protein Signaling 16) was also downregulated in many of the tests for either Gαi2 or Gβ2 (Figure 2). While RGS17 has been reported to act as a GTPase-activating protein (GAP) on free Gαi2 and Gαo under pre-steady-state conditions [44], it remains to be determined if the depletion of RGS16 in the Gαi2 and Gβ2 knockdown cells is biologically relevant.

### RNAi-based perturbations

The present studies were extended to reveal which signaling networks are associated with the reduction of the Gαi2 protein in the presence of lipopolysaccharide (LPS), Pam2CSK4 (Pam_2_) or prostaglandin E2 (PGE_2_). To observe the effect of the absence of Gαi2 in a ligand-dependent manner, gene expression was examined in both control RAW 264.7 cells (control lentiviral vector transfected cell lines) and Gαi2-deficient cells (Gαi2 shRNA-harboring lentiviral cell lines) after stimulation with LPS (100 ng/ml LPS and 100 pM LPS-binding peptide), Pam_2_ (350 nM) or PGE_2_ (10 µM) using 16K oligonucleotide microarrays. According to the criterion of a ≥2 fold change in expression, exposure of the cells to LPS for 30 min resulted in the up-regulation of nearly 100 transcripts in both control and Gαi2 knockdown cells in a similar pattern. A partial image of the set of clustered dendrograms is shown in Figure 3. Pam_2_ or PGE_2_ treatment of both cells also led to a very similar pattern of gene expression. However, there were some subtle differences in the changes in transcript levels induced by LPS or Pam_2_ treatment in the cells lacking the Gαi2 protein (Table 3). These genes include early growth response 2 (Egr2), the nuclear factor of kappa light polypeptide gene enhancer in B-cells inhibitor, zeta (Nfkbiz), tumor necrosis factor (TNF) and the 1810011O10 RIKEN clone, whose expressions were enhanced by LPS or Pam_2_ in Gαi2-deficient cells.

**Table 3:** Ligand-induced gene expression changes in Gαi2-deficient cells.

Gene	Assay	W vs Gi2-sh	LPS 0.5 h	Gi2: LPS 0.5 h	F: LPS	Pam2 0.5 h	Gi2: Pam2 0.5 h	F: Pam2	PGE 0.5 h	Gi2: PGE 0.5 h	F: PGE
Nfkbiz	Microarray	1.03	24.94	30.70	**5.76**	27.80	30.05	**2.25**	1.41	1.34	−0.07
	QRT-PCR		72.78	123.26	**50.48**	86.74	131.52	**44.79**	1.48	1.34	−0.14
Egr2	Microarray	0.60	12.11	18.10	**5.99**	14.80	20.74	**5.94**	0.71	0.78	0.14
	QRT-PCR		21.17	25.23	**4.06**	16.86	45.33	**28.47**	0.76	0.52	−0.62
1810011O10Rik	Microarray	1.01	4.81	10.16	**5.35**	4.00	8.63	**4.63**	1.42	1.34	−0.09
	QRT-PCR		53.51	84.18	**30.67**	71.06	116.53	**45.47**	3.45	2.32	−1.13
Tnf	Microarray	0.92	7.99	13.17	**5.18**	7.93	18.22	**10.29**	0.69	0.66	−0.06
	QRT-PCR		15.44	23.70	**8.26**	15.86	20.93	**5.07**	0.92	0.55	−0.73

Note: The numbers are in fold differences. F (F factor) was calculated by (ligand effect in knockdown) – (ligand effect in wild type): Ligand effect = 2^X^−1, if X≥0 or  = 1−1/2^X^, if X<0.

Where, X is the log_2_ ratio.

LPS is the major constituent of the outer membrane of Gram-negative bacteria. LPS binds to the cell surface receptor CD14, which enhances TLR4-dependent LPS recognition [45], [46], [47]. TLR4 activation engages a set of MyD88 (myeloid differentiation primary-response protein 88) adaptor family members, including MyD88, TIRAP (TIR domain containing adaptor protein), TRIF (TIR domain containing adaptor protein inducing IFNβ), and TRAM (TRIF related adaptor molecule). Pam_2_ is a synthetic diacylated lipopeptide, and these lipid-modified proteins are present in the cell membranes of bacterial cell walls. The intracytoplasmic signaling events associated with Pam_2_ stimulation are mainly engaged in TLR2/6 activation, which also leads to stimulation of the MyD88-dependent pathway [48]. Therefore, the synergistic effects of Gαi2 knockdown on LPS– or Pam_2_–induced Egr2, Nfkbiz or TNF (as shown in Table 3) suggest that some aspect of these signaling pathways is influenced by Gαi2 levels. This may reflect a heightened response or defective negative regulation in Gαi2-deficient cells. Consistent with the present results, stimulation of Gαi2-deficient peripheral T cells induced a hyper-responsive profile of interleukin-2, tumor necrosis factor, and interferon-gamma production [18]. Unlike Gαi1 or Gαi3, cells deficient in Gαi2 were reported to be hyper-responsive to cytokines, including IFN-gamma and IL-4 production following activation [20].

## Discussion

It is well established that siRNA efficacy is determined by how effectively the siRNA is incorporated into RISC [49], [50], [51]. This necessitates the testing of several candidate siRNAs to identify an effective reagent. Recently, it has been shown that testing of many siRNAs can be circumvented by *in vitro* digestion of a long dsRNA using the dicer enzyme. This produces a population of siRNAs covering a much larger region of target sequence and usually obviates the need to test multiple sequences for each gene. The caveats, however, are that this approach theoretically increases the possibility of an undesirable knockdown of other genes with significant homology to the target sequences used as the dicer template, and siRNAs derived from synthesized long dsRNAs by the T7 RNA polymerase system can trigger a potent induction of interferon α and β in a variety of cell lines due to the initiating 5′ triphosphate [34].

Three different silencing/delivery systems (siRNA, ASO and shRNA) and different sequences in the target genes in each case were used to assess how the knockdown of a particular gene may affect molecular mechanisms. The significant knockdown in the Gαi2 and Gβ2 protein levels suggests that the knockdown levels achievable with siRNA, ASO or shRNA may be sufficient to assess a transient or permanent phenotype [29].

‘Off-target effects’ can occur if the sequence identity between the siRNA and random mRNA transcripts is high enough to cause RNAi to knockdown the expression of non-targeted genes. However, in our experiments, sequence-specific off-target effects were not obvious except for the ASO designed for Gαi2, which indicates that the design of oligonucleotides is certainly important and should be tested in the genomic scale before it is used to knockdown a target gene in biological studies. Kim et al. [29] and Siolas et al. [52] found that synthetic RNA duplexes ∼27 nucleotides in length can be up to 100 times more potent than traditional 21-unit siRNA oligomers. Enhancing the potency of RNAi duplexes and thus lowering the effective concentration of that molecule is another preferable working direction to minimize off-target effects. The enhanced potency of the ∼27 nucleotide siRNA is attributed to the fact that it is diced by the dicer and directly delivered to the RNA-induced silencing complex (RISC).

Extracellular stimulants induce various cellular responses by regulating diverse pathways, and perturbation of any genes in the pathway can generate altered regulation of signaling networks. In a mouse model, *in vitro* stimulation of splenocytes with formalin-killed *Staphylococcus aureus* resulted in significantly increased production of IL1β, TNF, and IL12p40 in Gαi2 (−/−) compared to control mice [26]. Mice with targeted deletion of Gαi2 develop an inflammatory bowel disease closely resembling ulcerative colitis, and the IFNγ and IL1β levels were increased in the inflamed colons [53]. In RAW 264.7 cells, the expression level of Gαi2 was relatively high compared to that of Gαi3, whereas Gαi1 was not expressed (Table 1). Gαi2 proteins may play a role in modulating LPS-activated signaling through TLR4, leading to inflammatory mediator production in RAW 264.7 cells. There was a significant increase in the LPS-induced production of TNFα in Gαi2-deficient RAW 264.7 cells compared with control cells (Table 3). Subsequent QRT-PCR studies also confirmed that TNFα levels following a LPS challenge were significantly greater in Gαi2-deficient cells. This is consistent with the published data from Gαi2 (−/−) mice [26], [54].

Taken together, success was obtained with the knockdown of target genes in RAW 264.7 cells using chemically synthesized siRNA, lentiviral shRNA and ASO. The specificity of the different platforms of RNAi was also assessed through DNA microarrays, and the findings showed the successful development of siRNA reagents against the genes of interest and the useful application of vector-based RNAi to RAW 264.7 cells for functional studies. Well-designed RNAi would be a very useful tool to knockdown specific target genes and to study the functional roles of molecules within the relevant signaling network system.

## Materials and Methods

### Nucleic acid sequences for shRNAs, siRNAs and ASOs

The shRNAs, siRNAs and ASOs used in these studies were based on the following sequences: shRNAs (Gαi2-LV40: 5′-GTC TAC AGC AAC ACC ATC C-3′, Gαi2-LV11: 5′-GCA CAG AGT GAC TAC ATC C-3′, Gβ2-LV43 & 4: 5′-CAT CTG CTC CAT CTA TAG TC-3′), siRNAs (Gαi2-I: 5′-GAG CAA GTT TGA GGA TCT A-3′, Gαi2-L: 5′-CCG CTT ACT ACC TGA ATG A-3′, Gβ2-G: 5′-GGA CGG AAA GCT CAT CAT T-3′, Gβ2-H: 5′-GGA CGA CAA CCA AAT CAT C-3′) and ASOs (Gαi2: 5′-TTT AGA GCG CTC GGC TGC CG-3′, Gβ2: 5′-ATG AGC TTT CCG TCC TGG GA-3′).

### siRNAs or ASO treatments

All ASOs in this study were synthesized by ISIS Pharmaceuticals, and all siRNAs were synthesized and HPLC-purified by Qiagen Inc. (Valencia, CA). Briefly, RAW 264.7 cells in 24-well dishes were transfected with 400 nM of either ASO or siRNA using either FuGENE6 (Roche Molecular Biochemicals, Indianapolis, IN) or Lipofectamine 2000 (Invitrogen, Carlsbad, CA), respectively. For siRNA transfection, an incubation volume of 200 µl was used for 4 hr followed by the addition of media up to 1 ml. For ASO transfection, an incubation volume of 500 µl was used. siRNA transfections were repeated 24 hr after the initial transfection while a single transfection was done for ASO treatments. Cells were harvested at 48 hr post-transfection for isolation of the RNA or at 72 hr post-transfection for isolation of the protein.

### Virus construction

Lentiviral vectors were constructed and used for virus generation as described previously [5], [6]. Annealed shRNA linkers were ligated into a *Bam*H1/*Xho*1-digested pEN_mH1c plasmid. The cassette containing the mH1 promoter and the shRNA was subcloned to a lentiviral expression vector by site-specific recombination using the Gateway system (Invitrogen). The lentiviral vector contains the GFP gene driven by the ubiquitin promoter followed by an IRES (internal ribosome entry site) sequence and an antibiotic resistance gene for the selection of infected cells. The resultant lentiviral vectors were transfected into 293T cells two other plasmids referred to as ‘packaging’ and ‘envelope’ plasmids: pCMVΔR8.91 (expresses HIV *gag*, *pol* and *rev* genes), and pMD.G (expresses vesicular stomatitis virus G envelope protein), respectively. The packaging and envelope plasmids were a gift from the Didier Trono Lab, Geneva, Switzerland [55]. At 48 hr post-transfection, culture supernatants were collected and concentrated using Centricon Plus-80 units according to the manufacturer's instructions (Millipore Corporate, Billerica, MA). Virus was titrated by infecting 293T cells and assessing the percent of GFP positive cells using cytometric analysis 48 hr post-infection. The titered virus was used to infect RAW 264.7 cells at a MOI (multiplicity of infection) of five 293T-transducing units per RAW 264.7 cell, at virus concentrations of 1 to 5×10^7^/ml. These infection conditions routinely resulted in an average of 20% transduction efficiency, suggesting that a single productive viral integration occurs for every 25 viruses used on RAW 264.7 cells. Infected cells were selected according to their antibiotic resistance. These infection conditions routinely resulted in 10–25% transduction efficiency, suggesting that approximately one productive viral integration occurs for every 5 viruses used on RAW 264.7 cells. Infected cells were selected according to their antibiotic resistance.

### Western blotting

Cells were lysed with lysis buffer containing 150 mM NaCl, 50 mM NaF, 20 mM Tris, pH 7.5, 1% Triton X-100, 0.5% sodium deoxycholate, 0.1% SDS, and protease inhibitor mixture (Roche Diagnostics).

Thirty µg of protein was run on a NuPAGE 4–12% Bis-Tris gel (Invitrogen). Total protein was transferred to a nitrocellulose membrane (Schleicher and Schuell BioScience, Inc., Keene, NH) and was subsequently immunoblotted for the protein of interest. The membrane was blocked overnight at 4°C, which was followed by incubation with protein specific primary antibody for 2 to 3 hr at room temperature. After incubation with a secondary antibody conjugated to HRP (horseradish peroxidase) (Amersham Biosciences, Piscataway, NJ) for 1 hr at room temperature, the membrane was developed using a SuperSignal West Pico Chemiluminescent Substrate (Pierce Biotechnology, Inc., Rockford, IL) and exposed to X-OMAT film (Kodak).

### DNA microarray

Cells were cultured in media containing 0.5% FBS (for PGE_2_, S1P and nicotinic acid) or 10% FBS (for LPS, Pam_2_, Pam_3_, CpG, zymosan and polyIC) for 18 hr; stimulated with an agonist for 30 min, 2 hr or 4 hr; and were then harvested with Trizol (Invitrogen, Carlsbad, CA). Cells for the basic RNAi tool tests of siRNA, shRNA or ASO were cultured in media containing 10% FBS and were harvested with Trizol. Three micrograms of total RNAs from the cells were used as the starting material for the microarray analysis.

The 16K mouse oligonucleotide arrays were fabricated using an inkjet-printing method by Agilent Technologies (Palo Alto, CA). These oligo arrays includes 13,536 probes of 70mers (Operon Technologies Inc., Alameda, CA) and 2,304 probes of 65mers (Sigma-Genosys, The Woodlands, TX). The platform description (platform accession number GPL254) is available at GEO (Gene Expression Omnibus: http://www.ncbi.nlm.nih.gov/projects/geo/). The aminoallyl method was utilized for the preparation of the fluorescently labeled target samples.

### Quantitative RT-PCR

Quantitative real-time RT-PCR was performed using iCycler (Bio-Rad Laboratories, Inc., Hercules, CA) following the manufacturer's protocol. The measurement was normalized to a β actin RNA control. The primers of genes are as follows: Nfkbiz, F 5′-CGA TGG ACC GGT TTG CA-3′, R 5′-GTA GGC GTT TGC GGT GAT G-3′; Egr-2, F 5′-GTG CCA GCT GCT ATC CAG AAG, R 5′-GGC TGT GGT TGA AGC TGG AG-3′; TNF, F 5′-CCC TCA CAC TCA GAT CAT CTT CT-3′, R 5′-GCT ACG ACG TGG GCT ACA G-3′; β-actin, F 5′-CTT TGC AGC TCC TTC GTT GC-3′, R 5′-ACG ATG GAG GGG AAT ACA GC-3′. The remaining primer sequences are available upon request.

## Supporting Information

Table S1 Expression of selected signaling genes in RAW 264.7 cells, as assessed by Affymetrix GeneChips and RT-PCR. There were multiple probe sets in some cases. U: unavailable, P: present, A: absent, M: marginal.
(0.04 MB XLS)

Table S2 mRNA expression levels (log_2_ ratio) of G protein subunits assessed by DNA microarrays in shRNA, siRNA and ASO-treated RAW 264.7 cells.
(0.05 MB XLS)
